# Crisis resolution teams: are we doing things well?

**DOI:** 10.1192/j.eurpsy.2024.1268

**Published:** 2024-08-27

**Authors:** J. J. Martínez Jambrina, L. P. Gómez, A. M. G. Alvarez, C. P. Miranda, S. P. Alvarez, N. A. Alvargonzalez, I. F. Arias

**Affiliations:** Psychiatry, Hospital San Agustín, Avilés, Spain

## Abstract

**Introduction:**

Crisis resolution teams (CRTs) are a crucial component of mental health care, providing timely support to individuals experiencing acute mental health crises. This abstract delves into the concept of crisis and seeks to identify the patients who stand to benefit from these specialized services.

**Objectives:**

Defining crisis within the context of CRTs can be complex. It encompasses not only immediate emergencies but also broader mental health distress.

Research suggests that suitable candidates for CRT interventions are those facing acute mental health crises : This includes individuals experiencing suicidal ideation, severe agitation, or severe emotional distress.

La “Escala de Evaluación de Resolución de Crisis” (Crisis Resolution Team Assessment Tool, CRTAT) de Sonia Johnson es una herramienta diseñada para para medir la efectividad de los CRT y la duración de la intervención en crisis. Establece un límite de seis semanas como el período máximo durante el cual se debe ofrecer la atención en crisis.

Existen otras escalas de evaluación para medir la eficacia de la resolución de crisis:

1. **Escala de Intensidad de Crisis (CIS):** se utiliza para medir la gravedad de la crisis y la necesidad de intervención inmediata.
2. **Escala de Evaluación de Crisis de Brage Hansen (BCES):** se enfoca en la evaluación de crisis suicidas y evalúa la intensidad de la ideación suicida y la urgencia de la intervención.
3. **Escala de Evaluación de Crisis de Eriksson (ECAS):** Diseñada para evaluar la intensidad de la crisis en pacientes psiquiátricos, la ECAS se centra en la agitación, la ansiedad y la angustia emocional.

**Methods:**

- Studies have explored the effectiveness of CRTs and the perspectives of service users. Understanding how patients perceive crisis and CRT services is crucial for tailoring interventions effectively.

**Results:**

**Conclusions:**

- CRTs play a vital role in mental health care, offering timely support to individuals experiencing crises. While defining crisis is complex, suitable candidates often include those in acute distress requiring immediate intervention. Understanding the perspectives of service users and the diverse nature of crisis experiences informs effective crisis resolution strategies.

**Disclosure of Interest:**

None Declared

